# Challenges in detecting and diagnosing substance use in women in the acute psychiatric department: a naturalistic cohort study

**DOI:** 10.1186/s12888-016-1124-y

**Published:** 2016-11-17

**Authors:** Torill Vassli Sallaup, Arne Einar Vaaler, Valentina Cabral Iversen, Ismail Cuneyt Guzey

**Affiliations:** 1 Department of Tiller DPS, St Olavs University Hospital, Trondheim, Norway; 2 Department of Neuroscience, Faculty of Medicine, The Norwegian University of Science and Technology, Trondheim, Norway; 3 Department of Østmarka, St Olavs University Hospital, Trondheim, Norway

**Keywords:** Acute psychiatry, Substance use, Substance use disorders, Sex differences

## Abstract

**Background:**

This study examines sex differences in substance use and substance use disorder in the acute psychiatric department, and possible interactions between sex and clinical and social factors associated with this phenomenon.

**Methods:**

Data concerning substance use were collected in a naturalistic cohort study (*n* = 384, 51.6% male, 48.4% female) in an acute psychiatric department. Recent intake of substances at admission, diagnosis of substance use disorder and demographic and socioeconomic information were recorded. At admission, serum and urine samples were analysed for substance use and breath analysis was performed for alcohol levels.

**Results:**

Twice as many men as women were diagnosed with substance use disorder, whereas there were no gender differences in the number of positive toxicology screenings. Toxicology screening revealed the use of non-prescribed medication with addiction potential in 40% of both female and male patients many of whom did not report this in the admission interview. A low level of education in men and absence of parental responsibility in women showed a statistically significant interaction with a current diagnosis of substance use disorder.

**Conclusions:**

Despite no sex differences in positive toxicology screenings in the acute psychiatric department, twice as many men as women are diagnosed with substance use disorders. The use of prescription drugs with addiction potential was widely under-reported by both sexes, in patients with no prescriptions for the medications. Women with no parental responsibility are overrepresented among those diagnosed with substance use disorder, as are men with a low level of education.

**Trial registration:**

The study is registered with the ClinicalTrials.gov identifier NCT01415323

## Background

Concurrent substance use and substance use disorders are prevalent among patients in acute psychiatry [1–6]. Clinical studies have consistently found that people with mental illness and concurrent substance use disorders have a more severe course of illness [7], and also an increased risk of premature death [8]. Despite the high relevance of substance use occurring in combination with mental illness, substance use disorders are often reported to be under-detected in the psychiatric population [9–12]. People with both disorders are shown to have difficulties accessing help for either of the conditions [13]. Also, they are vulnerable to poorer treatment responses and outcomes in the long run [7, 10, 14, 15].

There is a discordance between the number of males and females who are undergoing addiction treatment. Women with substance use disorders are less likely to enter treatment compared with male substance users [16–18]. Globally, despite the fact that one out of three drug users is female, only one out of five in treatment is female [19]. In Norway, one out of two substance users is female; yet, females constitute only one third of the patients in Norwegian addiction treatment facilities [20]. The reason behind this gender discordance is uncertain, but may be related to both a less severe substance use in women that does not fill the criteria for a diagnosis, and also to social and structural barriers [16, 19].

Although there is still a male dominance in substance use [21, 22], women in developed countries have increased both the frequency and quantity of their substance use over recent decades [21–23]. The gender gap in substance use has narrowed particularly among the youngest cohorts [23]. Compared with men, women are found to be more vulnerable to adverse medical, psychiatric and social consequences from substance use [17]. With respect to the use of alcohol, cannabis and opioids, women are also found to be vulnerable to developing an accelerated progression from moderate use to substance use disorders [16, 24]. An exception to the observed pattern of male dominance in substance use is that a higher proportion of females use prescription drugs with addiction potential [3, 7, 25]. Women with mental illness also have particularly high comorbidity rates for sedative hypnotics and opioid use disorders resulting from prescription drugs as compared to men [26, 27].

Identification of substance use disorders creates the basis to plan addiction treatment. The first contact with specialised health care services for many patients with substance use combined with psychiatric disorders is the psychiatric acute and emergency services. It is important to evaluate the quality of this clinical examination and to investigate whether the reported gender-gap is present in these clinical settings.

One starting point for investigating the reasons behind the observed sex differences in the identification of substance use is the procedures for admissions to acute psychiatric departments. The diagnosis of substance use disorders in acute admissions is an ongoing process from admittance to discharge. Often, the diagnosis is established at the initial evaluation, based on self-reported information, physical examination and laboratory testing. The primary aim of this study was to examine sex differences in substance use and substance use disorder in the psychiatric acute department, and how sex interacts with possible clinical and social factors associated with this phenomenon.

## Methods

### Setting

The Norwegian acute psychiatric services are public and catchment area based. St Olavs University Hospital, Department of Acute Psychiatry, is situated in the county of Sør-Trøndelag in central Norway. All patients in the catchment area ≥ 18 years with acute psychiatric conditions in need of hospitalisations are admitted to this department. Patients with only substance use disorders are admitted to other, separate facilities. The basis for acute psychiatric hospitalisations is short-term admissions [3]. The median length of stay in the present data material was six days.

The patients were examined on arrival at the psychiatric department by the physician on duty, during the first day by specialist in psychiatry or in clinical psychology, and a number of times during in-patient stay by specialists or residents in psychiatry.

### Sample

Acutely admitted inpatients were consecutively asked to participate in a study where investigation of substance use was both part of the study procedure and the clinical routine in the department [28]. A total of 795 patients were admitted to the hospital during the study period, of which 424 (53.7%) were included in the study. Based on the predefined inclusion criteria, 14 patients (1.8%) were excluded, while 150 (18.9%) were discharged from the hospital before they could be asked to participate, and 204 (25.7%) refused to participate. Where respondents were admitted more than once, only data from the first admittance were included. Data from 384 unique respondents requiring hospitalisations between September 1^st^, 2011 and March 31^st^, 2012 were included in the material. Some patients in acute psychiatric departments have limited capacity to understand information about clinical studies at admission. To ensure that the patients understood the information, written, informed consent was collected by a specialist in psychiatry or clinical psychology the first day after admission. The study is registered with the ClinicalTrials.gov identifier NCT01415323. Ethical approval was obtained from the Regional Ethical Committee, Central Norway (2011/137).

### Measures

Primary measures were 1) recent intake of substances at admission and 2) diagnosis of substance use disorder. The physicians assessing recent intake of substances in the admission interview completed a study form with two questions, one on current drug influence, and, the other on substance use during the preceding week. The questions were formulated as follows: Based on all available information at the time of clinical assessment: 1) In your opinion, is this patient under the influence of drugs or alcohol at admission (tick off): Not at all, mildly, moderately, markedly, or uncertain? 2) Is there any information on recent substance intake (tick off): benzodiazepines, opiates, amphetamines, cannabis, cocaine, alcohol, others, or none? The physician based this evaluation on information from the referral form and by questioning the patients about recent use of the different substances. Breath analysis for estimation of blood alcohol levels was administered by staff during the admission interview (valid tests *n* = 244, 63.5%). Serum and urine samples were collected as soon as possible within the first 24 hours after admission (valid screens *n* = 342, 89%). Substances analysed in urine samples were alcohol, stimulants, opioids and cannabis, and in serum samples the substances were benzodiazepines and derivatives, z-drugs and opioids. On-site urine tests were not used due to the limited accuracy of the method [29]. Samples were analysed by the local clinical pharmacology laboratory using a liquid chromatography mass spectrometry (LC-MS) method. For most patients the results of these tests were not available to the clinicians at discharge.

Diagnoses according to ICD-10 criteria for research [30] were set at discharge in a weekly consensus meeting including the patients therapist and specialists (clinical psychologists or psychiatrists) of whom at least one had personally examined the patient. After discharge, the medical records for all the respondents were examined for all previous diagnoses of substance use disorders. Diagnoses of substance use disorders were categorised by: 1) prescription drugs (sedative hypnotics and opioids); and, 2) alcohol; and 3) illegal drugs (cannabis, stimulants and opioids).

Recent intake of substances at admission was measured by the following statements: 1) Respondent was assessed as influenced by substances during medical examination at admission (influence of substances at admission); or 2) if there were reported recent substance intake (reported recent substance intake); or 3) substance use was asserted by breath-, urine- or serum toxicology screens (positive toxicology screening).

The following baseline measures were included as covariates: demographic and socioeconomic information (sex, age by quartiles, employment, education (primary school, 10 years of schooling; high school, 13 years of schooling; or higher education, such as college or university degrees), parental responsibility for minors, and whether living with a partner). Also included as covariates were any previous hospitalisations in psychiatric departments, legal status of admission (voluntary/coerced), and previously diagnosed substance use disorder.

### Statistical analysis

Statistical analysis of the data was performed using the Statistical Package for the Social Sciences (IBM, SPSS, version 21). The study sample was described by sex and diagnoses of substance use disorders, and use of chi square tests for categorical variables. The continuous variable age was not normally distributed (skewed to the left) and was, therefore, categorised by quartiles.

We used a model built on crude analysis of substance use disorder by sex and the data describing the respondents. The interactions studied made use of statistically significant sex differences from chi square tests and was further analysed by logistic regression. The value that, on a theoretical base was assumed to have the lowest association with substance use disorders was used as a reference (e.g. parental responsibility, higher education and no previously diagnosed substance use disorder). *P*-values for women or men from the crude analysis ≤ .05 were analysed further in the adjusted logistic regression analysis (sex*statistical significant variables for women or men) to assess whether the relation between the specific factors and incidence of substance use disorder was overrepresented in women or men.

## Results

Clinical and social description of the sample by sex and substance use disorder is summarised in Table 1. More men than women diagnosed with substance use disorders in the current hospitalisation had parental responsibility for minors (27.4% and 8.9%, respectively) and had no more education than primary school (55.8% and 44.4.% respectively). The overrepresentation of men with substance use disorder and low education level was statistical significant in the adjusted analysis (*p* = .009), as was the female overrepresentation of substance use disorder if there was no parental responsibility (*p* < .000).

**Table 1:** Characteristics of respondents by sex and SUD

Characteristics	Total			Women			Men			Interactions
	SUD			SUD			SUD			
	Yes	No		Yes	No		Yes	No		
	*n* (%)	*n* (%)	*p* ^1^	*n* (%)	*n* (%)	*p* ^1^	*n* (%)	*n* (%)	*p* ^1^	*p*
Age, quartiles										
• 18–25	38 (27.1)	58 (23.9)		15 (33.3)	37 (26.2)		23 (24.2)	21 (20.6)		
• 26–37	38 (27.1)	59 (24.3)		14 (31.4)	35 (24.8)		24 (25.3)	24 (23.5)		
• 38–49	43 (30.7)	55 (22.6)		12 (26.7)	29 (20.6)		31 (32.6)	26 (25.5)		
• 50 +	21 (15)	71 (29.2)	.045	4 (8.9)	40 (28.4)	.067	17 (17.9)	31 (30.4)	.224	
Marital status										
• Living with a partner	25 (17.9)	69 (28.7)		8 (17.8)	38 (27.3)		17 (17.9)	31 (30.7)		
• Living alone	115 (82.1)	171 (71.3)	.144	37 (82.2)	101 (72.7)	.120	78 (82.1)	70 (69.3)	.420	
Parental responsibility										
• Yes	30 (21.4)	64 (26.3)		4 (8.9)	37 (28.1)		26 (27.4)	27 (26.5)		
• No	110 (78.6)	179 (73.7)	.048*	37 (91.1)	104 (71.9)	.013*	69 (72.6)	75 (73.5)	.887	<.001*
Education										
• Primary school	73 (52.1)	94 (39.2)		20 (44.4)	57 (41)		53 (55.8)	37 (36.6)		.001*
• High school	53 (37.9)	106 (44.2)		18 (40)	61 (43.9)		35 (36.8)	45 (44.6)		<.001*
• College/university	14 (10)	40 (16.7)	.029*	7 (15.6)	21 (15.1)	.806	7 (7.4)	19 (18.8)	.009*	-
Employment										
• Labour	38 (29.7)	46 (20.7)		12 (28.6)	29 (22.7)		26 (30.2)	17 (18.1)		
• Benefits/pension	90 (70.3)	176 (79.3)	.069	30 (71.4)	99 (77.3)	.533	60 (69.8)	77 (81.9)	.079	
Legal status, admission										
• Coercion	41 (29.3)	46 (19.2)		14 (31.1)	26 (18.8)		27 (28.4)	20 (19.8)		
• Voluntarily	99 (70.7)	193 (80.8)	.031*	31 (68.9)	112 (81.2)	.098	68 (71.6	81 (80.2)	.182	

*p* ^1^ estimated by Pearsons chi square tests. Interaction test by bivariate (a*b) logistic regression. Significance level *p* ≤ .05

* statistically significant *SUD* substance use disorder

Overall, there was male dominance in all the different measures of substance use (Table 2). The only exception was the equal distribution of physicians’ assessment of intoxication at admission (22% for women and 27% for men, *p* = .295) and distribution of positive toxicology screenings in breath, urine and serum that showed similar numbers between sexes (49% and 52%, *p* = .544). Male dominance in recent substance use at admission and previously and new diagnoses of substance use disorders was statistically significant.

**Table 2:** Recent use of substances and previous and current SUD between sexes

	Women *n* = 186	Men *n* = 198	Comparison between sexes	
Variable	*n* (%)	*n* (%)	*x* ^2^	*p*
Previously diagnosed SUD	35 (18.8%)	71 (35.9%)	13.369	< .001*
Assessed as influenced by substances at admission	41 (22%)	53 (26.8%)	1.095	.295
Reported recent substance intake	58 (28.8%)	99 (50)	18.413	< .001*
Positive toxicology screening	91 (48.9%)	103 (52)	.368	.544
Diagnosed SUD at discharge	44 (23.7%)	92 (46.5)	24.485	< .001*
• Alcohol use disorder	22 (11.8%)	38 (19.2)	4.610	.032*
• Prescription drug use disorder	7 (3.8%)	5 (2.5)	.019	.890
• Illegal drug use disorder	15 (7.6%)	49 (24.8)	20.329	< .001*

(%) within gender. Calculations of ratio. *x* ^2^ estimated by Pearson’s chi-squared test. Significance level *p* ≤ .05, * statistically significant. *SUD* substance use disorder

Positive toxicology screening, reported recent substance intake, and diagnoses of substance use disorders were almost identical for men (52%, 50% and 46.5%). By contrast, females received almost twice as many positive screening results with regard to reports on their recent substance intakes and diagnoses of substance use disorder (48.9%, 28.8%, and 23.7%).

Toxicology screening revealed the use of non-prescribed medication with addiction potential in 40% of both sexes (43.7% in women and 36.3% in men). Benzodiazepines constituted the largest group of positive test results for drugs without prescription for both women and men (Table 3).

**Table 3:** Positive screening of prescribed prescription drugs between sexes

Variable	Women		Men	
	Positive screening prescription drugs	Non- prescribed (% of positive tests)	Positive screening prescription drugs	Non- prescribed (% of positive tests)
At least one of the following	90 (48.4%)	38 (43.7%)	88 (43.4%)	33 (36.3%)
Benzodiazepine derivatives	59 (31.7%)	25 (42.4%)	60 (30.3%)	25 (41.6%)
Zopiclone	23 (12.4%)	9 (39.1%)	17 (8.6%)	4 (23.5%)
Morphine	3 (1.6%)	1 (33.3%)	2 (1%)	-
Codeine	5 (2.7%)	3 (60%)	6 (3%)	1 (16.6%)
Methadone	-	-	3 (1.5%)	3 (100)

()% within sex and within positive tests

## Discussion

The main finding in the present study is that in spite of similar percentages of positive toxicology screenings between women and men (48.9% and 52%, respectively), diagnoses of substance use disorders were twice as prevalent in men. The prevalence of diagnoses of substance use disorders in our study was in line with recent findings, which reported a prevalence of 30–65% of such diagnoses in acute psychiatry (1–6).

Fløvig et al. [3], in a study from the same catchment area, presented numbers for the gender distribution of recent substance intake in acute psychiatry, and, based on similar laboratory testing, reported a recent substance intake in 83% of females and 80% of males. They reported a positive diagnosis of substance use disorders in 50% of males, but only in 16% of the females. Compared with our study, the percentages of recent substance intakes prior to admission and diagnoses of substance use disorders in men were similar, but the rate for females with positive diagnoses of substance use disorders was lower, although the differences did not reach statistical significance. In both Fløvig et al. and the present study, when positive toxicology screening results were considered, there was a discordance between substance use disorder diagnoses and recent substance intake, which was especially pronounced in females. This may have been caused by substance use not fulfilling the ICD-10 criteria for research for substance use disorder, or a bias in diagnosing women with substance use disorder [16].

Another reason behind the discordance may be the different patterns of substance use. Use of medication with addiction potential was widely under-reported in both sexes, given that 40% of positive toxicology screenings were from patients with no prescriptions for the medications, and many without self-report of recent intake. The prevalence of non-prescribed use of medication with addiction potential has previously been reported to be 15–36% of positive toxicology screenings in acute psychiatry [3, 31]. Our study showed less agreement between self-report of recent substance intake and toxicological screenings in acute psychiatric settings than that earlier reported.

Social factors are known barriers for women in undergoing addiction treatment [16, 19]. The same barriers may be relevant in diagnosing substance use disorders in females in acute psychiatry. We found that parental responsibility for minors was three times more prevalent among men diagnosed with substance use disorders then women (27% and 9% respectively, *p* = .013). This may reflect the manner in which parental responsibility and substance use by females are interpreted by the acute psychiatric services and the stigma that surrounds the issue. Female patients themselves may inhibit the discovery of problematic substance use. Caring for children may result in a need for child-care if the patient accepts treatment, but it may also increase a risk of reactions from the child-welfare authorities. Parental responsibility can therefore be a barrier to admitting substance abuse by females, and also an impediment to the diagnosis of substance use disorders. Another possible explanation for differences between the sexes in diagnosis may be the biased opinion of the health care professionals regarding the role of males and females in parenting.

The stigma associated to substance use may be a hindrance for addiction treatment for women [32, 33]. Stigma may discourage women from admitting substance use and further raise an obstacle to the diagnosis of substance use disorders in women using acute psychiatric services.

The higher comorbidity of PTSD, eating disorders, depression and anxiety in women has been suggested as the reason prompting women to seek help primarily for conditions other than substance use [16]. The interpretation of the meaning of the substance use, both by the women themselves and by professionals in acute psychiatry, may influence the reporting of lower rates of diagnosed substance use disorders in women [16, 18]. The acute use of substances can be interpreted as a (destructive) way of handling the mental stress suffered by females in psychiatric crises, rather than as a problem inextricably connected with the use of the substance itself [16]. Women and men in acute psychiatry with positive evidence of recent substance use at admission may not always qualify for a diagnosis of substance use disorder. Moreover, we need to reveal the evidence of substance use in order to assess the issue, especially when dealing with non-prescribed prescription drugs.

### Strengths and limitations

Deficiencies may exist in the data. The study sample was of moderate size, which may limit generalisability. The inclusion of subjects in this study was based on informed consent. The possibility of implicit selection of respondents may be relevant in research on substance use, as we do not know whether non-consenting patients and patients in other centres would exhibit different characteristics on the variables of interest. Validated screening instruments for detection of problematic substance use were not used in the clinical interview and this may have led to the exclusion of potentially identifiable problematic substance use.

However, the use of consecutively admitted acute patients from one catchment area strengthens the generalisability of the study. The representativeness is also strengthened by the preclusion of exclusion criteria except with respect to language skills.

## Conclusions

Despite the fact that there were no gender differences in positive toxicology screenings, twice as many men as women were diagnosed with substance use disorder in the acute psychiatric department. Use of prescription drugs with addiction potential was widely under-reported in both sexes, from patients with no prescriptions for the medications. Women with no parental responsibility were overrepresented among those diagnosed with substance use disorder, as were men with a low level of education.

## Abbreviations

ICD: International classification of diseases
LC-MS: Liquid chromatography mass spectrometry method
PTSD: Post-traumatic stress disorder
SUD: Substance use disorder
